# Taxonomic Diversity, Predicted Metabolic Pathway, and Interaction Pattern of Bacterial Community in Sea Urchin *Anthocidaris crassispina*

**DOI:** 10.3390/microorganisms12102094

**Published:** 2024-10-20

**Authors:** Xinye Chen, Li Mo, Lin Zhang, Liyu Huang, Ziqing Gao, Jingjing Peng, Zonghe Yu, Xiaoyong Zhang

**Affiliations:** University Joint Laboratory of Guangdong Province, Hong Kong and Macao Region on Marine Bioresource Conservation and Exploitation, College of Marine Sciences, South China Agricultural University, Guangzhou 510642, China

**Keywords:** sea urchin, 16S rRNA gene, gut microbiota, *Anthocidaris crassispina*

## Abstract

Bacterial assemblages associated with sea urchin are critical to their physiology and ecology within marine ecosystems. In this study, we characterized the bacterial communities in wild sea urchin *Anthocidaris crassispina* captured in Daya Bay, South China Sea. A total of 363 amplicon sequence variants belonging to nine phyla and 141 genera were classified from intestine, body surface, and surrounding seawater samples. Proteobacteria, Firmicutes, and Bacteroidetes were the dominant bacteria phyla found in this study. A network analysis of bacterial interspecies interactions revealed varying complexity, stability, connectivity, and relationship patterns across the samples, with the most intricate network observed in the surrounding seawater. Metagenomic predictions highlighted the distinct bacterial metabolic pathways, with significant differences between intestine and seawater samples. Notably, pathways associated with polysaccharide degradation, including chitin derivatives, starch, and CoM biosynthesis, were markedly abundant, underscoring the gut microbiota’s key role in digesting algae. In addition, other metabolic pathways in intestine samples were linked to immune response regulation of sea urchins. Overall, this study provides a comprehensive overview of the bacterial community structure and potential functional roles in A. crassispina.

## 1. Introduction

As an ancient marine invertebrate, sea urchin includes over 900 species and thrives in marine benthic ecosystems worldwide [1]. Over the past century, it has served as a pivotal model organism in biotechnological research, contributing to studies on molecular mechanisms, gene engineering, cell cycle regulation, neurotoxicity, and toxicology [2,3,4]. Beyond its value in biological research, the sea urchin is crucial to marine ecosystems. It acts as an important indicator of environmental stress in marine settings and plays a key role in consuming marine algae, thus maintaining the ecosystems balance [5]. Additionally, sea urchins are popular seafood in some Southeast Asia countries [6], significantly contributing to the local aquaculture economies as a valuable source of aquatic organisms.

Recently, various novel marine products derived from sea urchins such as naphthazarin pigments, astaxanthin, and aromatic compounds have demonstrated with remarkable bioactive properties [7,8,9]. Furthermore, sea urchin coelomic fluid has shown significant antiviral and antioxidant potential [10,11]. In traditional Chinese medical science, sea urchin shells are considered a valuable remedy with potent anti-inflammatory properties [12]. However, emerging issues such as overfishing, climate change, and marine pollution have severely impacted sea urchin populations [13]. Conservation efforts should focus on promoting artificial urchin reproduction.

Previous research has highlighted the correlations between gut microbiome and its hosts [14]. Gut microorganisms participate in various metabolic processes, contributing significantly to the digestion and immune functions of sea urchins [15]. Furthermore, gut-derived probiotics have proven effective in promoting reproduction and disease management in aquatic species, such as shrimp and tilapia [16]. In sea urchins, the gut microbiota supports host growth by providing nitrogen and facilitating digestion [17]. It also contributes to the nutrient cycling of the marine environments by degrading intestinal digesta into highly nutritious food resources for many marine organisms [18]. Studying the gut microbiome of sea urchins could provide more efficient breeding strategies, discovering novel potential probiotics and controlling the disease outbreaks in sea urchin populations, reducing reliance on antibiotics and supporting sustainable aquaculture practices. Given that sea urchins host abundant and complex gut microbial communities, their gut microbiome efficiently reflects the status of the marine environment [19], making it highly relevant to aquaculture research. However, knowledge about the gut microbiome composition and its relations with marine environment in sea urchin *Anthocidaris crassispina* remains limited. To date, only a few sea urchin species have had their gut microbiome specifically studied, including *Abatus agassizii* [20], *Lytechinus variegatus* [21], *Stomopneustes variolaris*, *Tripneustes gratilla*, and *Diadema savignyi* [22].

Although most sea urchins are herbivorous, they can also feed on sediment [23], which allows them to ingest microorganisms from the sediments and introduce them into their intestines. Recent studies have revealed the diverse microbial communities in the marine sediment, closely related to the gut microbiota of a number of aquatic organisms [15]. Feeding on sediment and its impact on the gut microorganisms can influence the overall health and nutrition of sea urchins. By absorbing probiotics from sediments to intestines, sea urchins may enhance their digestion, immune system, and even reproduction [24]. However, mechanisms of microbial exchange between the surrounding sediment and urchin guts remain rarely studied. It has been found that the composition, diversity, and functional categories of gut microbiota can vary significantly among different sea urchin species, and the epibiotic microbiota in sea urchins is highly species-specific [25]. However, other external factors were found to be related with the alterations of the gut bacterial diversity and composition of sea urchin such as ocean warming, barren levels of hosts’ habitats, and their feeding diets [26,27,28]. 

In addition, the body surfaces of sea urchins host a high diversity of microorganisms [25]. Notably, the prevalence of *Vibrio* and *Exiguobacterium* on the body surfaces of diseased urchins has been found to significantly increase compared to healthy individuals [29]. This dysbiosis of the microbial community on the sea urchin’s body surface often leads to related diseases, which emphasize the role of body surface microbiota in identifying sea urchin health issues [30]. Such diseases result in increased mortality among sea urchins, leading to a significant loss in urchin production. Therefore, it is crucial to fully understand the mechanisms and develop effective treatments of the diseases [31]. Investigating the body surface microbiota of sea urchins provides a more comprehensive understanding of the normal epibiotic composition and the shifts in microbial communities triggered by various diseases [25]. In sea urchins, studies on microorganisms have primarily focused on gut, pharynx, and other tissues [20,22,32], knowledge of the external microbiota in sea urchins remains limited [33]. Moreover, urchins can derive specific microorganisms through horizontal transmission, making the microbial composition of surrounding water critical to maintaining the microbial homeostasis in sea urchins.

*A. crassispina* is a widely distributed and commonly cultured urchin species in China [34]. In this study, samples from the intestines, body surfaces, and seawater of sea urchin species *A. crassispina* were collected from Daya Bay, South China Sea, and the diversity of their bacterial communities was analyzed through high-throughput sequencing technology. A high diversity of bacterial 16S rRNA sequences was found in urchin-associated samples, providing insight into bacterial community in sea urchins through taxonomic annotation, community composition, diversity calculation, network analysis, and gene function prediction. Correlations between the surrounding seawater and the intestines of sea urchins were identified. Significant differences in bacterial diversity and richness were observed among all samples. Specific bacterial taxa in each sample and the core bacteria involved in the digestion process of sea urchins were discovered.

## 2. Materials and Methods

### 2.1. Sample Collection

**Figure 1:**
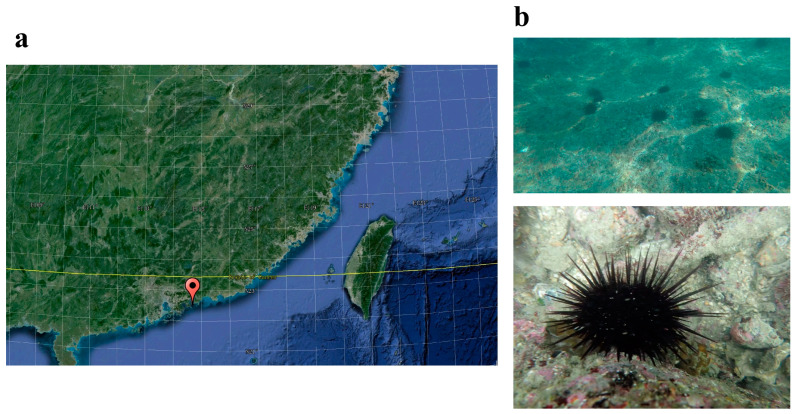
Map of the Daya Bay and the location of the urchin sampling site (**a**). Photos of *Anthocidaris crassispina* taken during sampling (**b**).

Adult sea urchins *A. crassispina* (*n* = 5) were collected off the coast of Daya Bay (114°41′5.70″ E, 22°33′6.36″ N) in the South China Sea at a depth of 10 m in May 2020 (Figure 1). Five sampling sites were selected, with an interval distance of more than 2 km between each site, and one urchin was collected from each site. After collection, sea urchins were stored at 4 °C in a tank containing in situ seawater. Seawater samples (0.5 L) were also collected from the surroundings of the sea urchins at each sampling site. These seawater samples were slowly vacuum-filtered separately through a 0.22 μm filter membrane for subsequent DNA extraction (EMD Millipore Corporation, Danvers, MA, USA). The filter membranes were then placed in 1.5 mL sterilized centrifuge tubes and stored in liquid nitrogen. Within 5 h of sampling, the body surfaces and intestines of *A. crassispina* were gently rinsed with nuclease-free sterile water and dissected using sterile scissors and placed in sterilized centrifuge tubes. A total of 15 samples (intestine, *n* = 5; body surface, *n* = 5; surrounding seawater, *n* = 5) were collected and kept frozen at −20 ℃ for later molecular analyses.

### 2.2. DNA Extraction and High-Throughput Sequencing

Each urchin sample was cut separately and soaked with 75% ethanol, then samples were washed three times with sterile seawater. Later DNA of each intestine and body surface samples were extracted using an DNeasy PowerSoil Kit (Qiagen, Hilden, Germany), DNA of each seawater sample was extracted using NucleoSpin Tissue Kit (MACHEREY-NAGEL). The V4–V5 region of 16S ribosomal RNA was amplified using the 515F and 907R bacterial primers [35].

PCR amplification was performed using 60 μL reactions that included 30 μL of High-Fidelity PCR Master Mix (New England Biolabs, Beverly, MA, USA), 0.4 μM each of forward and reverse primers, and approximately 20 ng of DNA templates. Subsequently to amplification, all PCR products were purified using the GeneJET Gel Extraction Kit (Thermo Scientific, Vantaa, Finland). The NEB Next^®^ DNA Sample Preparation Kit was utilized to construct 16S rRNA sequencing libraries, which were subsequently sequenced on the Illumina MiSeq platform (Version 3).

### 2.3. Sequences Analysis and Taxonomic Annotation

The raw paired-end sequences from each sample were acquired using the Illumina platform and subsequently imported into the QIIME2-2023.2 platform for analysis [36]. Firstly, raw sequences were demultiplexed into each group by DNA barcode, and primer and low-quality reads with an average quality score <20 were filtered by the QIIME2 demux command. Raw sequence data were demultiplexed and quality filtered using the q2 demux plugin followed by denoising with DADA2 [37]. The high-quality forward and reverse reads were then merged and correctly assigned to their respective samples using DNA barcodes [38]. Subsequently, the UCHIME plugin was employed for chimera detection and elimination, leading to the creation of a feature table for amplicon sequence variants (ASVs). Later, all ASVs were aligned by the qiime2-alignment function [39] and the phylogeny tree was constructed by q2-phylogeny function [40]. Taxonomic annotation was conducted by the qiime2-feature-classifier function [41] through the classify-sklearn naive Bayes taxonomy classifier against the Greengenes 13_8 99% OTUs reference sequences [42]. Metabolic functions were predicted by the “PICRUSt2” script under the Python 3 environment. The metagenome prediction result was later analyzed by “ggpicrust2” package in R [43]. The ko abundance was firstly converted to KEGG pathway abundance, and then the pathway differential abundance was analyzed using the ALDEx2 method. Finally, differential pathways between gut and surrounding seawater were selected and visualized.

### 2.4. Statistical Analysis

The biom-format document generated by QIIME2 was converted into a text file for the subsequent analysis of bacterial community diversities. Rarefaction curves, along with alpha and beta diversity for each sample, were calculated using Bray–Curtis metrics through the ‘GUnifrac’, ‘ggplot2’, and ‘vegan’ packages in RStudio [44,45]. The interspecific comparisons of each sample were executed by the ‘vegan’ and ‘ggplot2′ packages of Rstudio [46]. The later picturing drawing and analysis of bacterial diversity were conducted by the ‘pheatmap’, ‘ggpub’, and ‘circlize’ packages of Rstudio [47,48]. Statistical significance (*p* value) was calculated through univariate analysis (*t*-test). Co-occurrence network of bacterial interactions among all samples were analyzed through “Hmisc” package and later visualized by “Gephi” software (version 0.10) [49].

## 3. Results

### 3.1. Diversity of Bacterial Community in Sea Urchin

**Figure 2:**
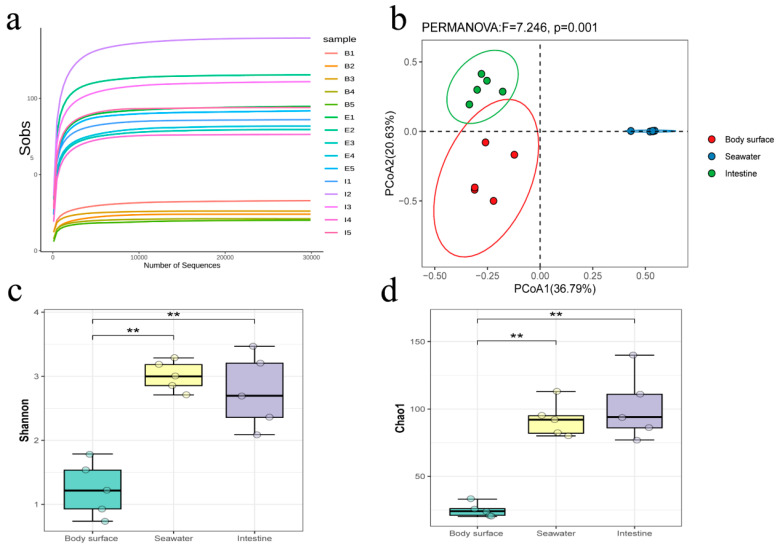
Rarefaction curves (**a**), principal co-ordinates analysis (**b**), Shannon index (**c**), and Chao1 index (**d**) of bacteria communities in the intestine, body surface, and surrounding seawater samples of sea urchin *A*. *crassispina*. ** *p* < 0.01.

A total of 15 samples were collected from the South China Sea and their bacterial communities were obtained through high-throughput sequencing technology based on the 16S rRNA gene. Amplicon sequencing results showed a total of 909,239 sequences, which ranged from 47,931 to 79,954 per sample, were obtained from all urchin samples, and the feature table containing 363 ASVs were generated from theses amplicon sequences. The rarefaction curves of each sample plateaued as sequence accumulation increased (Figure 2a), suggesting all samples could adequately reflect the bacterial communities observed in this study. β-diversity indicated distinct bacterial compositions among the different sample types (Figure 2b). The α-diversity analysis demonstrated that the body surface samples had a significantly lower bacterial diversity and richness compared to the other two sample groups (Wilcox test, *p* < 0.01), while no significant differences were observed between intestine and seawater samples (Figure 2c,d). These results indicate that bacterial communities in the intestine and seawater samples are more abundant and diverse than those on the body surface. Although their bacterial compositions were significantly different, the intestine and surrounding seawater samples showed no significant differences in microbial abundance and diversity.

### 3.2. Bacterial Taxonomic Classification and Community Composition

**Figure 3:**
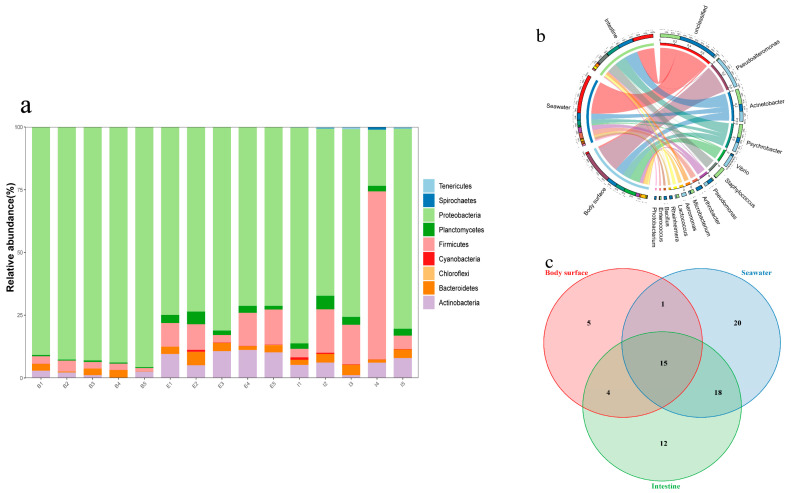
Relative abundance of bacteria at the phylum level (**a**), distribution of top 15 abundant bacteria genera (**b**), Venn diagram of bacteria at genera level (**c**), in the body surface, seawater, and intestine samples of sea urchin *A. crassispina* (B, body surface; E, environment seawater; I, intestine).

Bacterial taxonomic classification was performed on the q2-feature-classifier plugin. At the phylum level, a total of nine bacterial phyla were observed in the three types of samples (Table S1), including Proteobacteria, Firmicutes, Actinobacteria, Bacteroidetes, Planctomycetes, Cyanobacteria, Tenericutes, Spirochaetes, and Chloroflexi (Figure 3a). Among them, Proteobacteria, Firmicutes, and Bacteroidetes were the dominant bacterial phyla in this study, and Proteobacteria accounting for 66–94% of the bacterial communities as the most abundant bacteria. Firmicutes was the second most abundant phylum, constituting an average of 11.47% of the total bacterial communities. All phyla, except Chloroflexi, were found in all samples, while Chloroflexi were only observed in the seawater samples. Furthermore, based on the bacterial community composition, the abundances of Proteobacteria in the intestine and body surface samples were relatively higher than in the seawater samples, while Firmicutes and Actinobacteria were more abundant in the seawater and intestine samples compared to the body surface samples.

At the genus level, a substantial proportion of bacteria remained unclassified, accounting for 48.96% and 25.51% of the intestine and seawater samples, respectively (Figure 3b, Table S2). In the body surface samples, the dominant bacterial genera were *Pseudoalteromonas* (41.25%), *Vibrio* (15.49%), and *Acinetobacter* (12.98%). Apart from the unclassified bacteria in seawater and intestine samples, *Acinetobacter* was the most dominant bacteria genus in these two groups. The majority of *Pseudoalteromonas* (96.71%) and *Vibrio* (82.41%) detected in this study were distributed in the body surface samples, while *Staphylococcus* (96.99%) was mostly distributed in the intestine samples. In the seawater samples, nearly half of the bacteria remained unclassified at the genus level.

### 3.3. LefSe Analysis

**Figure 4:**
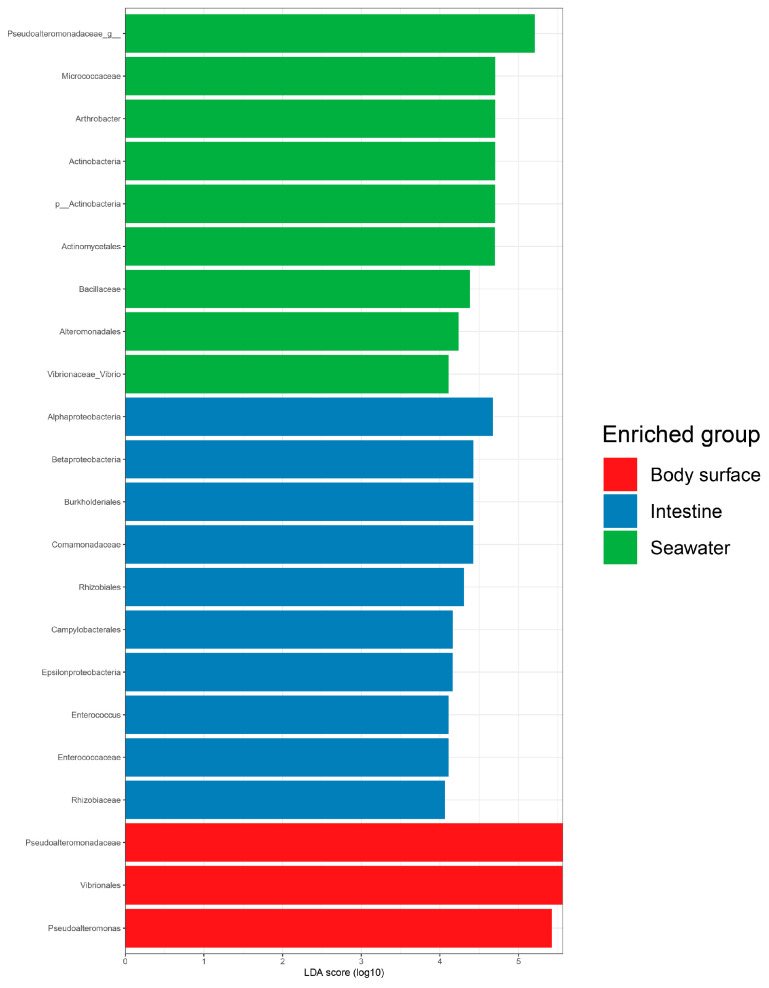
Potential biomarkers in each sample analyzed through LEfSe. Linear discriminant analysis (LDA) score was used to discover biomarkers in different ecosystem groups. The threshold value was log10 (LDA score) > 4.

The mutual and exclusive bacterial genera was clearly demonstrated by the Venn diagram (Figure 3c). As shown in the figure, a total of 15 bacteria genera were shared by all samples, with the seawater sample hosting the highest number of exclusive genera (20). The number of bacterial genera shared between the intestine and seawater samples (18) was much higher than that shared between the intestine and body surface samples (4), indicating the environmental microbiota may contribute more significantly to the composition of the urchins’ gut microbiota. Potential biomarkers that showed the greatest differences in bacterial communities among the different sample types were predicted by LefSe analysis (Figure 4). In the urchins’ body surfaces, Vibrionales and *Pseudoalteromonas* were the most differentially abundant taxa. Pseudoalteromonadaceae and *Arthrobacter* showed the most effecting size in seawater samples while the intestine samples presented Alphaproteobacteria, Rhizobiales, and Comamonadaceae.

### 3.4. Bacterial Gene Function Predictions of Sea Urchin

**Figure 5:**
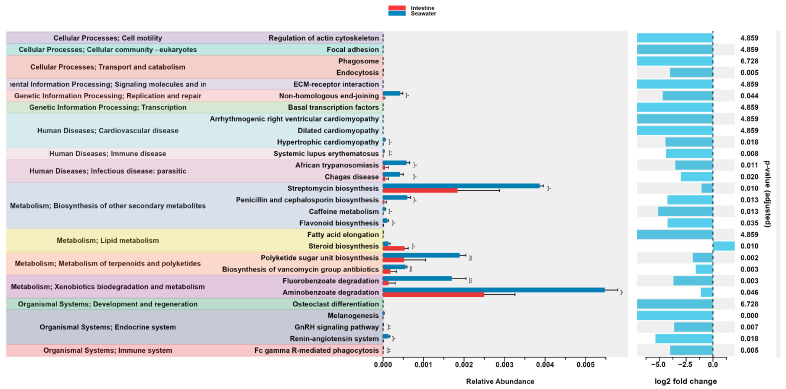
Results of the differential abundance analysis of predicted functional pathways in intestine and seawater samples. Error bar plots show a direct comparison of pathway abundance. Bar plots on the right represent log2 fold changes in the abundance of the pathways in different samples.

**Figure 6:**
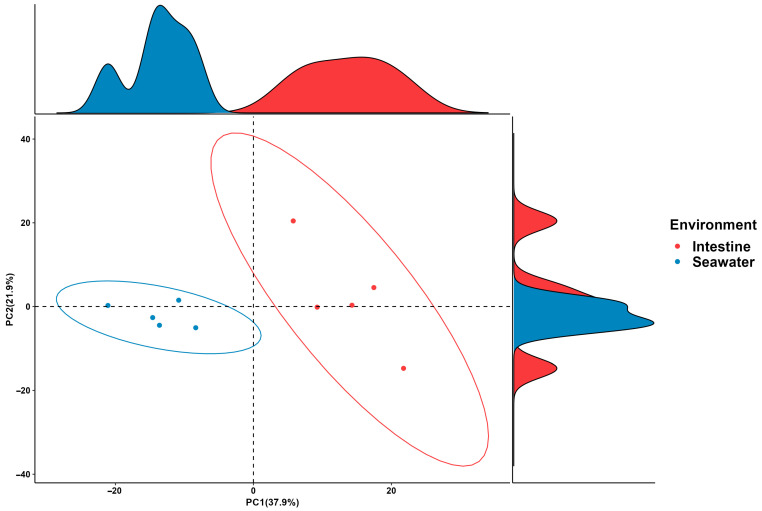
Principal component analysis (PCA) plot showing distances of predicted functional pathway abundances between intestine, body surface, and surrounding seawater sample.

**Figure 7:**
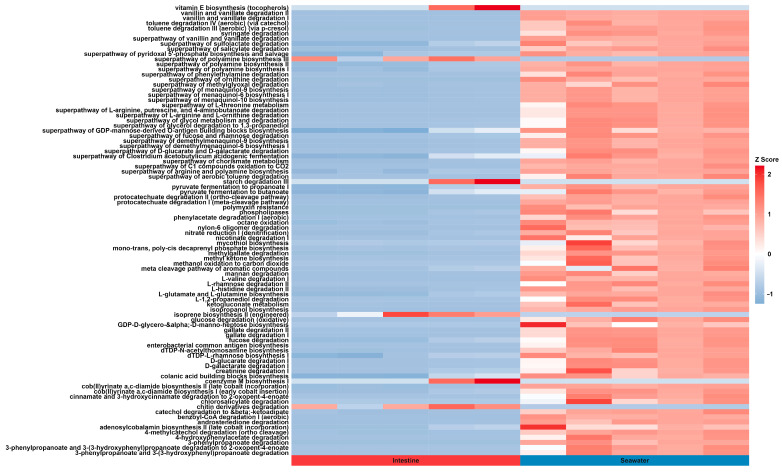
Heatmap of the significantly different predicted Metacyc pathways between intestine and surrounding seawater samples. Z score indicates the relative difference in one specific pathway in a sample from the average abundance level.

In this study, the metagenomics of bacterial communities were predicted using PICRUSt2 (v2.5.2) (Figure 5), revealing a wide range of gene functions and enzyme pathways. A total of 56 functions were predicted at KEGG pathway level 2, belonging to the 7 classes in KEGG pathway level 1, including proteins, cellular processes, environmental information processing, genetic information processing, human diseases, metabolism, and organismal systems. A principal component analysis (PCA) demonstrated noticeable differences in the compositions of predicted pathways between gut and surrounding seawater samples, which were consistent with PCA results of the bacterial community at the ASV level (Figure 6). Although there was no observed difference in the abundance of bacterial communities between intestine and surrounding seawater samples, a significant number of differential KEGG pathways (28) and Metacyc (89) pathways were identified (Figure 7). Predicted pathways including the superpathway of polyamine biosynthesis, starch degradation, isoprene biosynthesis, coenzyme M biosynthesis, chitin derivatives degradation, and steroid biosynthesis were notably more abundant in the intestine samples compared to the surrounding seawater.

### 3.5. Network Pattern of Bacterial Interaction

**Figure 8:**
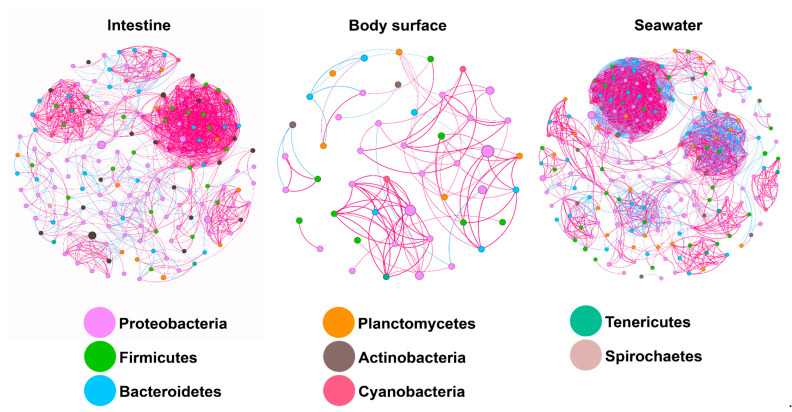
Co-occurring network of bacterial communities at amplicon sequence variant (ASV) level in the intestine, body surface, and seawater samples of sea urchin. Each node represents one bacterial ASV, and they are colored based on the phylum taxonomic annotation. Red edges represent positive correlations and bule edge represent negative correlation.

The co-occurrence pattern networks elucidate the interactions of bacterial interspecies among the body surface, intestine, and surrounding seawater of sea urchin *A. crassispina* (Figure 8). Among the three types of samples, the network pattern of surrounding seawater sample hosted the highest number of nodes (229) and edges (2272) and highest average degree (19.834), with relatively low positive correlations (71.57%), modularity (0.617), and density (0.087) among all edges. In the intestine sample, the network pattern consisted of 178 nodes, 1437 edges, and an average degree of 16.146, with 87.13% positive correlations, a modularity of 0.622, and a density of 0.091. The body surface sample had the lowest number of nodes (47) and edges (104) and the lowest average degree, but showed the highest positive correlations (88.46%), modularity (0.759), and density (0.096). These topological characteristics comprehensively illustrated the complexity, stability, connectivity, and interaction patterns in the three types of samples.

## 4. Discussion

In this study, a total of 909,239 bacterial 16S rRNA sequences were discovered in the seawater, intestines, and body surface samples of sea urchin *A. crassispina* through high-throughput technology. Sequences detected in the intestine and surrounding seawater samples showed a significantly higher abundance compared to the body surface sample, but no significant difference between the intestine and seawater samples. As many sea urchins ingest marine sediment as an alternative carbon source, the microbial community hosted in marine sediment may be selectively transplanted into the gut of sea urchin and contribute to the urchin digestion process. This could explain the similarity in bacterial abundance between the intestine and surrounding seawater samples.

The bacterial taxonomic annotation revealed the composition of bacterial communities in each sample. The most dominant bacterial phylum in all samples was Proteobacteria, a frequently observed group in the marine environment, especially the intestines of sea urchins. Proteobacteria plays an essential role in the marine ecosystem, closely associated with hydrogen oxidation, sulfate reduction, and denitrification [50]. Previous studies have reported the relatively high abundance of Proteobacteria in the gut of sea urchins. These bacteria are involved in nutrient absorption, breaking down complex molecules, and may play a role in protecting sea urchins against pathogens. Firmicutes was the second most dominant phylum found in intestine samples of this study. Similarly, a previous study also reported Firmicutes as a major bacterial phylum in one algivorous sea urchin *Tripneustes gratilla* [51], and discussed its potential roles in the urchin food digestion and fermentation process due to its outstanding cellulolytic activity in the decomposition of plant biomass [27,52,53]. However, this hypothesis has not yet been verified.

As the major consumers of algae in the marine ecosystem [54], sea urchins lack the innate gut digestive enzymes to facilitate the breakdown of insoluble structural carbohydrates [21,55]. Moreover, a previous study has demonstrated the significant role of the gut microbiome in assisting with the digestion of complex compounds in sea urchins [56]. It is speculated that, due to the extreme conditions in the intestines of sea urchins (low pH and oxygen concentration, high CO₂ concentration), their gut microbiota is influenced by strong deterministic forces that shape its composition and function to compensate for the innate digestive limitations, such as deficiencies in polysaccharide degradation [51]. Additionally, it has been reported that the dominant bacterial phyla in red seagrass are Proteobacteria and Firmicutes [57], which is also the top two abundant bacterial phyla detected in urchin intestine samples of this study (with relative abundance of 64.11% and 23.42%, respectively). This coincidence may be attributed to active microbial transmission through sea urchin ingestion, which aids in algal polysaccharide degradation through bioactive polysaccharide-degrading enzymes.

At the genus level, we identified several bacterial genera that are rarely found in the gut microbiota of other urchin species, and compositions of dominant bacterial genera in this study were different from previous research (Table S3), indicating the species-specific bacterial communities and their distinct roles in sea urchins. In this study, *Acinetobacter*, *Psychrobacter*, and *Staphylococcus* were the dominant bacteria in the intestine samples. Among these, nearly all *Staphylococcus* in this study were found in the intestine samples, suggesting urchins may acquire this group of bacteria from their parental generation through vertical transmission. Although *Staphylococcus* is commonly recognized as a pathogenic bacterium that causes various diseases by attenuating the immune response [58], it has also been commonly found in the gut microbiota and slightly effect the gut microbial composition and health status of their hosts. Recent studies have reported a relatively high abundance of *Staphylococcus* in the body surface of sea urchin *S. intermedius* has been associated with the infection of red spotting diseases [59], but their role in the intestine tissues of sea urchin still remains unclear*. Acinetobacter* was the most abundant genus in the intestine samples in this study, and it is inferred that this bacterial genus may play a role in managing environmental stressors of the gastrointestinal systems and potentially influence the gut epithelium [60]. Moreover, one *Acinetobacter* strain isolated from marine sediments was found to have the remarkable ability of producing agarase (Leema Roseline and Sachindra, 2016), which may potentially contribute to the agal digestion process of sea urchins. However, the specific roles of *Acinetobacter* in the intestines of urchins still lack further investigation. The second most abundant genus in the intestine samples was *Psychrobacter.* As a cold-resistant bacterium commonly found in the marine environment, *Psychrobacter* was able to improve the native microbial diversity in the gastrointestinal of grouper *Epinephelus coioides* [61], but it was rarely found in the urchins’ gut tissues in the former investigations.

LEfSe analysis demonstrated the significant effect size of certain key bacterial taxa contributing to microbial community, clearly illustrating the specific bacteria in each sample. In the body surface samples, where a relatively lower abundance of bacteria was observed in this study compared to the other two sample types, only Vibrionales and *Pseudoalteromonas* were identified as potential biomarkers. Notably, nearly half of *Pseudoalteromonas* and Vibrionales detected in this study were found in the body surface samples, and the potential roles of these two bacterial genera in sea urchins have been verified in previous studies. A previous study conducted on the body surface bacterial community of the sea urchin *Strongylocentrotus intermedius* discovered the highest abundance of the *Pseudoalteromonas* in the healthy urchins, and a significantly higher abundance of *Vibrio* (32.47%) compared to the healthy urchins was also detected in the disease urchins suffered from red spotting disease. Moreover, previous studies found the bacteria *Pseudoalteromonas* and *Vibrio* could form biofilms that provide suitable substrate to induces larval settlement and metamorphosis of urchin [62,63]. Larval settlement is of significant importance to the survival and distribution of sea urchin, as it indicates the termination of the planktonic larval phase and start of a benthic adult phase. This process involved larva selection and colonization of suitable benthic habitat, and subsequently metamorphosis to adapt to a benthic lifestyle [64].

Additionally, it has been reported that the bacterial community of urchin body surface may significantly impact their health through nutrient cycling [33]. Previous researches had reported the occurrence of Alphaproteobacteria, Comamonadaceae, Rhizobiales, Campylobacterales as the key taxa in the sea urchin *A. agassizii*, *Lytechinus variegatus*, and *Tripneustes gratilla*, respectively [20,26,51,65]. *Enterococcus* was also detected in both the stomach and intestine tissues of sea urchin *Paracentrotus lividus* through the traditional cultural method with a relative higher abundance. Although the gut microbiota has been described as highly species-specific and variable [25], the intestine-specific bacteria identified in this study have already been observed with significant dominance in the gut tissues of other urchin species. However, the specific roles of these bacteria in the gut tissues still require further investigation.

The result of a PCA at the taxonomic level and predicted functions level had illustrated the differences in bacterial communities of different samples in this study. As shown in Figure 5, bacterial communities from body surface and intestine groups were plotted separately from those in the seawater group, and the PERMANOVA results revealed that the bacterial communities in seawater were significantly different from the intestine and body surface groups (*p* < 0.01). Furthermore, the Venn diagram showed the seawater sample shared a much broader bacterial community with the intestine sample (33) compared to the body surface sample (16), indicating that sea urchin intestine tends to recruit microorganisms from the sediment from surrounding seawater through ingestion, while the body surface of sea urchin, in spite of directly exposed to the surrounding seawater, shown a less inclination.

This result may be attributed to the significantly lower bacterial abundance and diversity in the body surface of sea urchin. The hydrophobic body surface of sea urchin could effectively reduce the affinity between the microbial cell membranes and the surface, thereby inhibiting colonization [66]. Moreover, urchin epibiotic microbiome plays a substantial role in preventing infections from external microbial pathogens. Invasions by other bacteria can lead to the dysbiosis of the epibiotic microbiome, resulting in severe diseases such as balding disease and spotting disease [25,31,33]. Therefore, the bacterial composition on the body surface is less likely to be affected by direct contact with microorganisms in the surrounding seawater. In contrast, the intestines of sea urchins host a more abundant and diverse bacterial community, and microorganisms can be easily transferred from the surrounding seawater or sediments to the intestine through ingestion [28]. Hence, although the body surface is constantly exposed to the surrounding seawater, the bacterial community on the sea urchin body surface shares fewer common bacteria with the surrounding seawater compared to the intestine.

The network pattern delineated the interactions among bacterial species within each sample, revealing relationships such as mutualism and competition in the bacterial community, as well as the complexity and stability of the bacterial interactions. As shown in Figure 8, the Proteobacteria showed the most abundant among all networks, highlighting its significance in the bacterial interactions of sea urchins. The body surface sample exhibited a relatively lower bacterial abundance compared to other samples, resulting in a simpler network pattern as evidenced by the minimal number of nodes and edges. Although no significant difference was detected between the intestine and seawater samples, the number of nodes and edges and the average degree in the seawater sample were much higher than those in the intestine sample, indicating a more stable and complex network pattern in the surrounding seawater. In addition, the positive correlations were generally higher than the negative correlations, showing a mutualistic interaction pattern in the bacterial community. However, the positive correlations in seawater sample were much lower than the intestine sample, suggesting a higher antagonistic interaction among seawater bacterial species.

The bacterial potential pathways were predicted through the PICRUST2 script. Previous research had revealed the lack innate gut digestive enzymes in the sea urchins, and their remarkable seagrass consuming ability may be attributed to the synthesis of complex carbohydrates degradation and essential biomolecules for protein and lipid incorporation by various gut bacterial communities [67]. PCA result showed the distinct variance in the predicted pathway between the intestine and seawater samples, with differential pathways also observed between these two sample types. While most of predicted pathways in both the KEGG and Metacyc databases showed higher abundances in the surrounding seawater sample, several predicted pathways demonstrated significant higher abundance in the intestine sample. Two polysaccharide degradation-correlated pathways in the intestine, chitin derivative degradation, and starch degradation were found to significant more abundant than the sediment sample, reflecting the intensive algal polysaccharide digestion process and the significant role of gut microbiota in the intestine of sea urchin. Moreover, the CoM biosynthesis pathway was found to have potential correlations with the polysaccharide degradation. The efficiency of polysaccharide degradation could influence the availability of substrates of methanogenesis, indirectly affecting the demand for CoM in methane-producing pathways [68]. Another abundant pathway in the intestine sample was the isoprene biosynthesis pathway. It is reported that the isoprene plays an essential role in interacting with the host’s immune system through affecting the behavior of gut cell, thereby regulating intestinal health and disease [69]. However, knowledge on the mechanism of isoprene affecting sea urchin intestine remains limited.

## 5. Conclusions

In summary, this study revealed the bacterial community profiles of the intestine, body surface, and surrounding seawater in sea urchin *A. crassispina*. A diverse bacterial community was detected in each sample, their community composition, interspecies interactions, and predicted metagenomic pathways were obtained, and differences between each sample were analyzed. The bacterial community in the seawater sample was found to be significantly divergent from the other two groups of samples, and the number of bacteria in seawater sample shared with the intestine sample was much higher than the body surface samples. The network analysis of bacterial interspecies interaction showed the different levels of complexity, stability, connectivity, and relationship pattern in each sample, with the most stable and complex network pattern hosted by the surrounding seawater sample. The bacterial metagenomic prediction result revealed the potential metabolic pathways of bacteria in each sample, and the intestine and seawater sample displayed significant different pathway compositions. Significant differences in predicted pathways including chitin derivatives degradation, starch degradation, and CoM biosynthesis were detected using the ALDEx2 method, and their potential correlations with the polysaccharide degradation were discovered. However, there are a variety of predicted pathways that still remain unclear in the gut of sea urchins, and further investigation about the digestion mechanisms of sea urchin should be conducted in the future.

## Data Availability

Dataset available on request from the authors.

## Supplementary Materials

The following supporting information can be downloaded at: https://www.mdpi.com/article/10.3390/microorganisms12102094/s1, Table S1: Relative abundance of bacterial phyla in body surface (B), intestine (I) and surrounding seawater (E) samples of sea urchin *Anthocidaris crassispina*; Table S2: Mean relative abundance of bacterial phyla in each sample of sea urchin *Anthocidaris crassispina*; Table S3: Relative abundance of dominant bacterial taxa in gut of different urchin species. Refs [17,20,22,70] are cited.

## References

[1] Littlewood D.T.J., Smith A.B. (1995). A Combined Morphological and Molecular Phylogeny for Sea Urchins (Echinoidea: Echinodermata). Philos. Trans. R. Soc. Lond. Ser. B Biol. Sci..

[2] Hernández-Zulueta J., Rubio-Bueno S., Zamora-Tavares M.D.P., Vargas-Ponce O., Rodríguez-Troncoso A.P., Rodríguez-Zaragoza F.A. (2024). Metabarcoding the Bacterial Assemblages Associated with Toxopneustes Roseus in the Mexican Central Pacific. Microorganisms.

[3] Di Bernardo M., Di Carlo M. (2017). The Sea Urchin Embryo: A Model for Studying Molecular Mechanisms Involved in Human Diseases and for Testing Bioactive Compounds. Sea Urchin—From Environment to Aquaculture and Biomedicine.

[4] Adonin L., Drozdov A., Barlev N.A. (2021). Sea Urchin as a Universal Model for Studies of Gene Networks. Front. Genet..

[5] Buñuel X., Alcoverro T., Boada J., Zinkunegi L., Smith T.M., Barrera A., Casas M., Farina S., Pérez M., Romero J. (2023). Indirect Grazing-Induced Mechanisms Contribute to the Resilience of Mediterranean Seagrass Meadows to Sea Urchin Herbivory. Oikos.

[6] Suckling C.C., Zavell M.D., Byczynski A.L., Takeda B.T. (2022). Assessing the Potential of the Unexploited Atlantic Purple Sea Urchin, *Arbacia punctulata*, for the Edible Market. Front. Mar. Sci..

[7] Pagliara P., De Benedetto G.E., Francavilla M., Barca A., Caroppo C. (2021). Bioactive Potential of Two Marine Picocyanobacteria Belonging to *Cyanobium* and *Synechococcus* Genera. Microorganisms.

[8] Cirino P., Brunet C., Ciaravolo M., Galasso C., Musco L., Vega Fernández T., Sansone C., Toscano A. (2017). The Sea Urchin Arbacia Lixula: A Novel Natural Source of Astaxanthin. Mar. Drugs.

[9] Guo Z.K., Wang R., Chen F.X., Liu T.M., Yang M.Q. (2018). Bioactive Aromatic Metabolites from the Sea Urchin-Derived Actinomycete *Streptomyces spectabilis* Strain HDa1. Phytochem. Lett..

[10] Salas-Rojas M., Galvez-Romero G., Anton-Palma B., Acevedo R., Blanco-Favela F., Aguilar-Setién A. (2014). The Coelomic Fluid of the Sea Urchin *Tripneustes Depressus* Shows Antiviral Activity against Suid Herpesvirus Type 1 (SHV-1) and Rabies Virus (RV). Fish Shellfish Immunol..

[11] Soleimani S., Mashjoor S., Mitra S., Yousefzadi M., Rezadoost H. (2021). Coelomic Fluid of Echinometra Mathaei: The New Prospects for Medicinal Antioxidants. Fish Shellfish Immunol..

[12] Jiao H., Shang X., Dong Q., Wang S., Liu X., Zheng H., Lu X. (2015). Polysaccharide Constituents of Three Types of Sea Urchin Shells and Their Anti-Inflammatory Activities. Mar. Drugs.

[13] Takami H., Won N., Kawamura T. (2013). Impacts of the 2011 Mega-Earthquake and Tsunami on Abalone *Haliotis discus hannai* and Sea Urchin *Strongylocentrotus nudus* Populations at Oshika Peninsula, Miyagi, Japan. Fish. Oceanogr..

[14] Macke E., Tasiemski A., Massol F., Callens M., Decaestecker E. (2017). Life History and Eco-Evolutionary Dynamics in Light of the Gut Microbiota. Oikos.

[15] Li J., Chen S., Wu P., Zhu C., Hu R., Li T., Guo Y. (2023). Insights into the Relationship between Intestinal Microbiota of the Aquaculture Worm *Sipunculus Nudus* and Surrounding Sediments. Fishes.

[16] Mohan K., Ravichandran S., Muralisankar T., Uthayakumar V., Chandirasekar R., Seedevi P., Rajan D.K. (2019). Potential Uses of Fungal Polysaccharides as Immunostimulants in Fish and Shrimp Aquaculture: A Review. Aquaculture.

[17] Hakim J.A., Schram J.B., Galloway A.W.E., Morrow C.D., Crowley M.R., Watts S.A., Bej A.K. (2019). The Purple Sea Urchin *Strongylocentrotus purpuratus* Demonstrates a Compartmentalization of Gut Bacterial Microbiota, Predictive Functional Attributes, and Taxonomic Co-Occurrence. Microorganisms.

[18] Sauchyn L.K., Scheibling R.E. (2009). Degradation of Sea Urchin Feces in a Rocky Subtidal Ecosystem: Implications for Nutrient Cycling and Energy Flow. Aquat. Biol..

[19] Hagstrom G.I., Levin S.A. (2017). Marine Ecosystems as Complex Adaptive Systems: Emergent Patterns, Critical Transitions, and Public Goods. Ecosystems.

[20] Schwob G., Cabrol L., Poulin E., Orlando J. (2020). Characterization of the Gut Microbiota of the Antarctic Heart Urchin (Spatangoida) *Abatus agassizii*. Front. Microbiol..

[21] Hakim J.A., Koo H., Kumar R., Lefkowitz E.J., Morrow C.D., Powell M.L., Watts S.A., Bej A.K. (2016). The Gut Microbiome of the Sea Urchin, *Lytechinus variegatus*, from Its Natural Habitat Demonstrates Selective Attributes of Microbial Taxa and Predictive Metabolic Profiles. Fems Microbiol. Ecol..

[22] Yao Q., Yu K., Liang J., Wang Y., Hu B., Huang X., Chen B., Qin Z. (2019). The Composition, Diversity and Predictive Metabolic Profiles of Bacteria Associated With the Gut Digesta of Five Sea Urchins in Luhuitou Fringing Reef (Northern South China Sea). Front. Microbiol..

[23] Hollertz K. (2002). Feeding Biology and Carbon Budget of the Sediment-Burrowing Heart Urchin *Brissopsis lyrifera* (Echinoidea: Spatangoida). Mar. Biol..

[24] Thompson B.A., Riddle M.J. (2005). Bioturbation Behaviour of the Spatangoid Urchin *Abatus Ingens* in Antarctic Marine Sediments. Mar. Ecol. Prog. Ser..

[25] Rodríguez-Barreras R., Dominicci-Maura A., Tosado-Rodríguez E.L., Godoy-Vitorino F. (2023). The Epibiotic Microbiota of Wild Caribbean Sea Urchin Spines Is Species Specific. Microorganisms.

[26] Brothers C.J., Van Der Pol W.J., Morrow C.D., Hakim J.A., Koo H., McClintock J.B. (2018). Ocean Warming Alters Predicted Microbiome Functionality in a Common Sea Urchin. Proc. R. Soc. B.

[27] Masasa M., Kushmaro A., Kramarsky-Winter E., Shpigel M., Barkan R., Golberg A., Kribus A., Shashar N., Guttman L. (2021). Mono-Specific Algal Diets Shape Microbial Networking in the Gut of the Sea Urchin *Tripneustes gratilla elatensis*. Anim. Microbiome.

[28] Park J.-Y., Jo J.-W., An Y.-J., Lee J.-J., Kim B.-S. (2023). Alterations in Sea Urchin (*Mesocentrotus nudus*) Microbiota and Their Potential Contributions to Host According to Barren Severity. NPJ Biofilms Microbiomes.

[29] Federico S., Glaviano F., Esposito R., Tentoni E., Santoro P., Caramiello D., Costantini M., Zupo V. (2023). The “Bald Disease” of the Sea Urchin *Paracentrotus lividus*: Pathogenicity, Molecular Identification of the Causative Agent and Therapeutic Approach. Microorganisms.

[30] Li R., Dang H., Huang Y., Quan Z., Jiang H., Zhang W., Ding J. (2020). Vibrio Coralliilyticus as an Agent of Red Spotting Disease in the Sea Urchin *Strongylocentrotus intermedius*. Aquac. Rep..

[31] Shaw C.G., Pavloudi C., Crow R.S., Saw J.H., Smith L.C. (2024). Spotting Disease Disrupts the Microbiome of Infected Purple Sea Urchins, *Strongylocentrotus purpuratus*. BMC Microbiol..

[32] Rodríguez-Barreras R., Tosado-Rodríguez E.L., Godoy-Vitorino F. (2021). Trophic Niches Reflect Compositional Differences in Microbiota among Caribbean Sea Urchins. Peerj.

[33] Brink M., Rhode C., Macey B.M., Christison K.W., Roodt-Wilding R. (2019). Metagenomic Assessment of Body Surface Bacterial Communities of the Sea Urchin, *Tripneustes gratilla*. Mar. Genom..

[34] Ding J., Chang Y., Wang C., Cao X. (2007). Evaluation of the Growth and Heterosis of Hybrids among Three Commercially Important Sea Urchins in China: *Strongylocentrotus nudus*, *S. intermedius* and *Anthocidaris crassispina*. Aquaculture.

[35] Chen X., Wang Y., Hou Q., Liao X., Zheng X., Dong W., Wang J., Zhang X. (2024). Significant Correlations between Heavy Metals and Prokaryotes in the Okinawa Trough Hydrothermal Sediments. J. Hazard. Mater..

[36] Bolyen E., Rideout J.R., Dillon M.R., Bokulich N.A., Abnet C.C., Al-Ghalith G.A., Alexander H., Alm E.J., Arumugam M., Asnicar F. (2019). Reproducible, Interactive, Scalable and Extensible Microbiome Data Science Using QIIME 2. Nat. Biotechnol..

[37] Callahan B.J., McMurdie P.J., Rosen M.J., Han A.W., Johnson A.J.A., Holmes S.P. (2016). DADA2: High-Resolution Sample Inference from Illumina Amplicon Data. Nat. Methods.

[38] Chen X., Liao X., Chang S., Chen Z., Yang Q., Peng J., Hu W., Zhang X. (2024). Comprehensive Insights into the Differences of Fungal Communities at Taxonomic and Functional Levels in Stony Coral *Acropora intermedia* under a Natural Bleaching Event. Mar. Environ. Res..

[39] Katoh K., Misawa K., Kuma K., Miyata T. (2002). MAFFT: A Novel Method for Rapid Multiple Sequence Alignment Based on Fast Fourier Transform. Nucleic Acids Res..

[40] Price M.N., Dehal P.S., Arkin A.P. (2010). FastTree 2–Approximately Maximum-Likelihood Trees for Large Alignments. PLoS ONE.

[41] Bokulich N.A., Kaehler B.D., Rideout J.R., Dillon M., Bolyen E., Knight R., Huttley G.A., Gregory Caporaso J. (2018). Optimizing Taxonomic Classification of Marker-Gene Amplicon Sequences with QIIME 2’s Q2-Feature-Classifier Plugin. Microbiome.

[42] McDonald D., Price M.N., Goodrich J., Nawrocki E.P., DeSantis T.Z., Probst A., Andersen G.L., Knight R., Hugenholtz P. (2012). An Improved Greengenes Taxonomy with Explicit Ranks for Ecological and Evolutionary Analyses of Bacteria and Archaea. ISME J..

[43] Yang C., Mai J., Cao X., Burberry A., Cominelli F., Zhang L. (2023). Ggpicrust2: An R Package for PICRUSt2 Predicted Functional Profile Analysis and Visualization. Bioinformatics.

[44] Oksanen J., Kindt R., Legendre P., O’Hara B., Stevens M.H.H., Oksanen M.J., Suggests M. (2007). The Vegan Package. Community Ecol. Package.

[45] R Core Team (2013). R: A Language and Environment for Statistical Computing.

[46] Wickham H. (2011). Ggplot2. Wiley Interdiscip. Rev. Comput. Stat..

[47] Gu Z., Gu L., Eils R., Schlesner M., Brors B. (2014). “Circlize” Implements and Enhances Circular Visualization in R. Bioinformatics.

[48] Kolde R., Kolde M.R. (2015). Package ‘Pheatmap’. R Package.

[49] Bastian M., Heymann S., Jacomy M. Gephi: An Open Source Software for Exploring and Manipulating Networks. Proceedings of the International AAAI Conference on Web and Social Media.

[50] Zhou Z., Tran P.Q., Kieft K., Anantharaman K. (2020). Genome Diversification in Globally Distributed Novel Marine Proteobacteria Is Linked to Environmental Adaptation. ISME J..

[51] Masasa M., Kushmaro A., Nguyen D., Chernova H., Shashar N., Guttman L. (2023). Spatial Succession Underlies Microbial Contribution to Food Digestion in the Gut of an Algivorous Sea Urchin. Microbiol. Spectr..

[52] Flint H.J., Bayer E.A., Rincon M.T., Lamed R., White B.A. (2008). Polysaccharide Utilization by Gut Bacteria: Potential for New Insights from Genomic Analysis. Nat. Rev. Microbiol..

[53] Thomas F., Hehemann J.-H., Rebuffet E., Czjzek M., Michel G. (2011). Environmental and Gut Bacteroidetes: The Food Connection. Front. Microbiol..

[54] Carnell P.E., Ierodiaconou D., Atwood T.B., Macreadie P.I. (2020). Overgrazing of Seagrass by Sea Urchins Diminishes Blue Carbon Stocks. Ecosystems.

[55] Lawrence J.M., Lawrence A.L., Watts S.A., Lawrence J.M. (2013). Chapter 9—Feeding, Digestion and Digestibility of Sea Urchins. Developments in Aquaculture and Fisheries Science.

[56] Becker P.T., Samadi S., Zbinden M., Hoyoux C., Compère P., De Ridder C. (2009). First Insights into the Gut Microflora Associated with an Echinoid from Wood Falls Environments. Cah. Biol. Mar..

[57] Singh R.P., Reddy C.R.K. (2014). Seaweed–Microbial Interactions: Key Functions of Seaweed-Associated Bacteria. FEMS Microbiol. Ecol..

[58] Fournier B., Philpott D.J. (2005). Recognition of *Staphylococcus Aureus* by the Innate Immune System. Clin. Microbiol. Rev..

[59] Wang L., He B., Chang Y., Ding J. (2020). Characterization of the Bacterial Community Associated with Red Spotting Disease of the Echinoid *Strongylocentroyus intermedius*. Aquaculture.

[60] Glover J.S., Browning B.D., Ticer T.D., Engevik A.C., Engevik M.A. (2022). Acinetobacter Calcoaceticus Is Well Adapted to Withstand Intestinal Stressors and Modulate the Gut Epithelium. Front. Physiol..

[61] Yang H.-L., Sun Y.-Z., Ma R.-L., Li J.-S., Huang K.-P. (2011). Probiotic *Psychrobacter* sp. Improved the Autochthonous Microbial Diversity along the Gastrointestinal Tract of Grouper *Epinephelus coioides*. J Aquac Res Dev..

[62] Huggett M.J., Crocetti G.R., Kjelleberg S., Steinberg P.D. (2008). Recruitment of the Sea Urchin *Heliocidaris Erythrogramma* and the Distribution and Abundance of Inducing Bacteria in the Field. Aquat. Microb. Ecol..

[63] Peng L.-H., Liang X., Xu J.-K., Dobretsov S., Yang J.-L. (2020). Monospecific Biofilms of *Pseudoalteromonas* Promote Larval Settlement and Metamorphosis of *Mytilus coruscus*. Sci. Rep..

[64] Doll P.C., Caballes C.F., Hoey A.S., Uthicke S., Ling S.D., Pratchett M.S. (2022). Larval Settlement in Echinoderms: A Review of Processes and Patterns. Oceanogr. Mar. Biol..

[65] Green G.B.H., Hakim J.A., Chen J.-W., Koo H., Morrow C.D., Watts S.A., Bej A.K. (2021). The Gut Microbiota of Naturally Occurring and Laboratory Aquaculture *Lytechinus variegatus* Revealed Differences in the Community Composition, Taxonomic Co-Occurrence, and Predicted Functional Attributes. Appl. Microbiol..

[66] Mckenzie J.D., Grigolava I.V. (1996). The Echinoderm Surface and Its Role in Preventing Microfouling. Biofouling.

[67] Reisky L., Préchoux A., Zühlke M.-K., Bäumgen M., Robb C.S., Gerlach N., Roret T., Stanetty C., Larocque R., Michel G. (2019). A Marine Bacterial Enzymatic Cascade Degrades the Algal Polysaccharide Ulvan. Nat. Chem. Biol..

[68] Wu H.-H., Pun M.D., Wise C.E., Streit B.R., Mus F., Berim A., Kincannon W.M., Islam A., Partovi S.E., Gang D.R. (2022). The Pathway for Coenzyme M Biosynthesis in Bacteria. Proc. Natl. Acad. Sci. USA.

[69] Perez-Gil J., Rodriguez-Concepcion M. (2013). Metabolic Plasticity for Isoprenoid Biosynthesis in Bacteria. Biochem. J..

[70] Haditomo A.H.C., Yonezawa M., Yu J., Mino S., Sakai Y., Sawabe T. (2021). The Structure and Function of Gut Microbiomes of Two Species of Sea Urchins, *Mesocentrotus nudus* and *Strongylocentrotus intermedius*, in Japan. Front. Mar. Sci..

