# Supplementary material for: Taxonomic Diversity, Predicted Metabolic Pathway, and Interaction Pattern of Bacterial Community in Sea Urchin Anthocidaris crassispina
Source: Microorganisms. 2024 Oct 20;12(10):2094. doi: 10.3390/microorganisms12102094 (PMC11514596; doi:10.3390/microorganisms12102094)
Supplement: Supplementary file 1 [file microorganisms-12-02094-s001.zip › microorganisms-3259025-supplementary.pdf]

Taxonomic Diversity, Predicted Metabolic Pathway, and Interaction Pattern of Bacterial Community in Sea Urchin *Anthocidaris crassispina*

Xinye Chen, Li Mo, Lin Zhang, Liyu Huang, Ziqing Gao, Jingjing Peng, Zonghe Yu * and Xiaoyong Zhang *

University Joint Laboratory of Guangdong Province, Hong Kong and Macao Region on Marine Bioresource Conservation and Exploitation, College of Marine Sciences, South China Agricultural University, Guangzhou 510642, China

* Correspondence: yuzh@scau.edu.cn (Z.Y.); zhangxiaoyong@scau.edu.cn (X.Z.)

Table S1 Relative abundance of bacterial phyla in body surface (B), intestine (I) and surrounding seawater (E) samples of sea urchin *Anthocidaris crassispina*

phylum	Actinobacteria	Bacteroidetes	Chloroflexi	Cyanobacteria	Firmicutes	Planctomycetes	Proteobacteria	Spirochaetes	Tenericutes
B1	2.9544%	2.6917%	0.0000%	0.0115%	3.0196%	0.5349%	90.7496%	0.0000%	0.0383%
B2	2.2305%	0.3064%	0.0000%	0.0000%	4.4219%	0.3747%	92.6666%	0.0000%	0.0000%
B3	1.1806%	2.5980%	0.0000%	0.0000%	2.6068%	0.6150%	92.9997%	0.0000%	0.0000%
B4	0.0000%	3.2225%	0.0000%	0.0000%	2.5108%	0.4417%	93.8250%	0.0000%	0.0000%
B5	2.6208%	0.1204%	0.0000%	0.0062%	1.2232%	0.3821%	95.6472%	0.0000%	0.0000%
I1	5.2434%	2.0295%	0.0000%	0.9961%	3.3198%	2.1460%	86.0605%	0.0000%	0.2046%
I2	6.1695%	3.4434%	0.0000%	0.4687%	17.2299%	5.4123%	66.6226%	0.1004%	0.5532%
I3	1.1704%	4.1476%	0.0000%	0.1335%	15.7289%	3.1274%	74.8347%	0.0000%	0.8575%
I4	6.1248%	1.2257%	0.0000%	0.0513%	66.9910%	2.1575%	22.4304%	0.9618%	0.0575%
I5	8.0002%	3.3490%	0.0000%	0.1099%	5.3683%	2.7509%	79.7176%	0.0824%	0.6216%
E1	9.6088%	2.8022%	0.0000%	0.0042%	9.4613%	3.2165%	74.8873%	0.0000%	0.0197%
E2	5.1027%	5.2473%	0.1051%	0.6898%	10.2482%	5.0485%	73.5552%	0.0000%	0.0033%
E3	10.7368%	3.2205%	0.0000%	0.0827%	2.9994%	1.8145%	81.0769%	0.0000%	0.0692%
E4	11.1163%	1.5039%	0.0000%	0.0827%	13.2127%	2.7804%	71.3040%	0.0000%	0.0000%
E5	10.2236%	2.7964%	0.0926%	0.1451%	13.9866%	1.4546%	71.3011%	0.0000%	0.0000%

Table S2 Mean relative abundance of bacterial phyla in each sample of sea urchin *Anthocidaris crassispina*

genus	Body surface	Seawater	Intestine
Acidovorax	0.0000%	0.0000%	1.9534%
Acinetobacter	12.9768%	9.0526%	19.2946%
Aeromonas	5.7818%	0.0000%	0.0000%
Agrobacterium	0.0000%	0.0000%	0.1512%
Algoriphagus	0.0000%	0.1439%	0.0000%
Anaerospora	0.0080%	0.0000%	0.0895%
Arcobacter	0.0023%	0.0000%	0.0284%
Arthrobacter	0.0000%	7.5218%	0.6306%
Bacillus	0.0000%	3.4147%	0.0000%
Bacteriovorax	0.0000%	0.0092%	0.0000%
Brevundimonas	0.0000%	0.2489%	0.8911%
Candidatus	0.0077%	0.0000%	0.0000%
Cellvibrio	0.0000%	0.0115%	0.3806%
Chelativorans	0.0000%	0.2553%	0.1461%
Clostridiisalibacter	0.0000%	0.0156%	0.0000%
Clostridium	0.0000%	0.5571%	0.1123%
Cobetia	0.0000%	0.2960%	0.0000%
Comamonas	0.0000%	0.8732%	0.0000%
Coxiella	0.0000%	0.0000%	0.0090%
Cytophaga	0.4102%	0.0048%	0.4024%
Defluviitalea	0.0000%	0.0000%	0.3815%
Delftia	0.0000%	0.0000%	0.3069%
Desulfitispora	0.0000%	0.0105%	0.0084%
Enterococcus	0.6204%	0.1315%	1.8893%
Enterovibrio	0.0008%	0.0000%	0.0000%
Epulopiscium	0.0000%	0.1499%	0.0000%
Flavobacterium	0.0046%	0.7767%	1.0884%
Flectobacillus	0.0000%	0.1862%	0.0000%
Fusibacter	0.0000%	0.0000%	0.1308%
unclassified	2.2538%	48.9621%	25.5062%
Gloeobacter	0.0000%	0.0000%	0.0075%
Hoeflea	0.0000%	0.1028%	0.0000%
Hydrocoleum	0.0000%	0.0532%	0.0000%
Lactococcus	1.5848%	0.0312%	3.3220%
Limnobacter	0.0000%	0.2139%	0.2832%
Lysobacter	0.0000%	0.1213%	0.1261%
Massilia	0.0000%	0.0659%	0.0000%
Methylotenera	0.0000%	0.1410%	0.0000%
Microbacterium	1.7353%	0.9528%	4.2502%
Microbulbifer	0.0000%	0.0000%	0.2740%
Mucilaginibacter	0.0000%	0.1897%	0.0126%

Nocardioides	0.0000%	0.1735%	0.0036%
Novosphingobium	0.0973%	0.0000%	0.1512%
Paracoccus	0.0000%	0.4931%	0.0186%
Pedobacter	0.0000%	0.1156%	0.0000%
Phaeobacter	0.0000%	0.0105%	0.0081%
Photobacterium	0.0000%	2.1863%	0.0000%
Planctomyces	0.0911%	0.1105%	0.2925%
planctomycete	0.0000%	0.0000%	0.1773%
Planctomycete	0.0000%	0.0000%	0.0802%
Pseudoalteromonas	41.2523%	0.0516%	1.0450%
Pseudomonas	4.9369%	6.9450%	1.1599%
Pseudorhodobacter	0.0000%	0.6545%	0.1895%
Psychrobacter	12.0362%	6.5681%	16.9511%
Ralstonia	0.0000%	0.0099%	0.0000%
Reyranella	0.0000%	0.3900%	0.0413%
Rheinheimera	0.4351%	2.8958%	0.3042%
Rhizobium	0.0000%	0.8143%	0.6060%
Rhodobacter	0.0000%	0.3938%	0.0000%
Rhodococcus	0.0000%	0.1362%	0.5665%
Roseomonas	0.0092%	0.0000%	0.0000%
Ruegeria	0.0000%	0.3282%	0.0000%
Ruminococcus	0.0027%	0.0000%	0.0096%
Runella	0.0088%	0.0000%	0.0000%
Sphingobacterium	0.0000%	0.0108%	0.0000%
Sphingobium	0.0000%	0.4727%	0.0000%
Sphingopyxis	0.0000%	0.0525%	0.0000%
Staphylococcus	0.0000%	0.4864%	14.7348%
Stenotrophomonas	0.0000%	0.0000%	0.6048%
Synechococcus	0.0011%	0.0041%	0.0000%
Tenacibaculum	0.0000%	0.0000%	0.0096%
Tepidibacter	0.2168%	0.0764%	0.5036%
Vibrio	15.4914%	2.0230%	0.6824%
Vogesella	0.0349%	0.0115%	0.0293%
Xanthobacter	0.0000%	0.0926%	0.1548%

Table S3 Relative abundance of dominant bacterial taxa in gut of different urchin species

Urchin species	Top 1 bacterial taxa	Top 2 bacterial taxa	Top 3 bacterial taxa	Top 4 bacterial taxa	Top 5 bacterial taxa	reference
Anthocidaris crassispina	unclassified (25.51%)	*Acinetobacter* (19.29%)	*Psychrobacter* (16.95%)	*Staphylococcus* (14.73%)	*Microbacterium* (4.25%)	This study

Strongylocentrotus purpuratus	*Psychromonas* (32.94%)	*Propionigenium* (9.19%)	*Arcobacter* (8.83%)	*Sulfurimonas* (5.32%)	*Tissierella* (3.67%)	[1]
S. intermedius	Psychromonadaceae (16-53%)	Flavobacteriaceae (7-32%)	Rhodobacteraceae (1-10%)	Colwelliaceae (0.2-9%)		[2]
Abatus agassizii Guillaume	Bacteroidia (37.2%)	Gammaproteobacteria (36.1%)	Planctomycetacia (9.3%)			[3]
Stomopneustes variolaris	*Propionigenium* (45.81%)	*Prolixibacter* (15.72%)	*Photobacterium* (9.91%)	*Vibrio* (3.74%)	*Ferrimonas* (2.67%)	[4]

Reference

1. Hakim, J.A.; Schram, J.B.; Galloway, A.W.E.; Morrow, C.D.; Crowley, M.R.; Watts, S.A.; Bej, A.K. The Purple Sea Urchin *Strongylocentrotus Purpuratus* Demonstrates a Compartmentalization of Gut Bacterial Microbiota, Predictive Functional Attributes, and Taxonomic Co-Occurrence. *Microorganisms* **2019**, *7*, 35, doi:10.3390/microorganisms7020035.
2. Haditomo, A.H.C.; Yonezawa, M.; Yu, J.; Mino, S.; Sakai, Y.; Sawabe, T. The Structure and Function of Gut Microbiomes of Two Species of Sea Urchins, *Mesocentrotus Nudus* and *Strongylocentrotus Intermedius*, in Japan. *Front. Mar. Sci.* **2021**, *8*, 802754, doi:10.3389/fmars.2021.802754.
3. Schwob, G.; Cabrol, L.; Poulin, E.; Orlando, J. Characterization of the Gut Microbiota of the Antarctic Heart Urchin (Spatangoida) *Abatus Agassizii*. *Front. Microbiol.* **2020**, *11*, 308, doi:10.3389/fmicb.2020.00308.
4. Yao, Q.; Yu, K.; Liang, J.; Wang, Y.; Hu, B.; Huang, X.; Chen, B.; Qin, Z. The Composition, Diversity and Predictive Metabolic Profiles of Bacteria Associated With the Gut Digesta of Five Sea Urchins in Luhuitou Fringing Reef (Northern South China Sea). *Front. Microbiol.* **2019**, *10*, 1168, doi:10.3389/fmicb.2019.01168.
